# Improvement of Deep Brain Stimulation in Dyskinesia in Parkinson's Disease: A Meta-Analysis

**DOI:** 10.3389/fneur.2019.00151

**Published:** 2019-02-25

**Authors:** Yun Liu, Feng Li, Hansheng Luo, Qiuguang He, Lifen Chen, Yuan Cheng, Wenbin Zhang, Zongyi Xie

**Affiliations:** ^1^Department of Neurosurgery, The Second Affiliated Hospital, Chongqing Medical University, Chongqing, China; ^2^Department of Neurology, The Second Affiliated Hospital, Chongqing Medical University, Chongqing, China; ^3^Department of Functional Neurosurgery, Nanjing Brain Hospital Affiliated to Nanjing Medical University, Nanjing, China

**Keywords:** Parkinson's disease, deep brain stimulation, subthalamic nucleus, globus pallidus interna, dyskinesia

## Abstract

**Background:** Deep brain stimulation (DBS) of the subthalamic nucleus (STN) or globus pallidus internus (GPi) have been proven to be equally effective in improving motor-symptoms for advanced Parkinson's disease (PD) patients. However, it is unclear that which target stimulation is more effective in reducing dyskinesia. We conducted the meta-analysis to evaluate the efficacy of STN and GPi-DBS in the dyskinesia.

**Methods:** A systematic search was performed in PubMed, Embase, and the Cochrane Library databases. Controlled trials about the dyskinesia comparing the efficacy of GPi and STN DBS were included. Clinical data of dyskinesia and levodopa equivalent doses (LED) were collected for the meta-analysis.

**Results:** Eight eligible trials containing a total of 822 patients were included in this meta-analysis. Our results showed that GPi DBS offered a greater reduction of dyskinesia than STN DBS at 12 months after surgery, with an overall pooled SMD of 0.32 (95% CI = 0.06 to 0.59, *P* = 0.02). Treatment of STN DBS was associated with a greater reduction of LED compared with GPi DBS, with a change score of −320.55 (95% CI = −401.36 to −239.73, *P* < 0.00001).

**Conclusion:** GPi DBS is superior to reduce dyskinesia than STN DBS at 12 months after surgery for advanced PD patients. Further studies should focus on the different mechanism for dyskinesia reduction by GPi or STN DBS.

## Introduction

Parkinson's disease (PD) is a chronic and neurodegenerative disorder which affects 1% of the population over 60 years old (1). Dopamine replacement therapy remained the most effective symptomatic treatment of PD since Levodopa was first introduced for the treatment with PD in the 1960s. However, dopaminergic therapies are eventually associated with motor fluctuations and levodopa-induced dyskinesia. In a community-based study, the mean times of onset of dyskinesia were 6.6 years (2). Other studies have reported that 50% of PD patients experienced dyskinesia after 5 years from introduction of L-dopa (3), and this percentage up to 95% after 15 years of therapy (4). The clinical manifestations of the dyskinesia included head, hand, foot, body, and trunk of involuntary movement. General types of dyskinesia could be divided into peak-dosed dyskinesia (PDSK), diphasic dyskinesia (DDSK), and off-period dystonia according to the course of the disease, clinical manifestation, and the relationship with medicine. As the curative effect decreased gradually, off-period dystonia appeared in the early morning or night, resulting in leg and foot cramp (5). Different types of dyskinesia were observed in PD patients. PDSK, off-period dystonia and DDSK were accounted for about 80, 30, and 20%, respectively. Furthermore, different types of dyskinesia could appear or appear alternately in the same patient at the same time (6). Previous studies have shown that incidence of dyskinesia was positively correlated with following factors, including youth, women, long course of levodopa treatment, high dose levodopa treatment and low weight (7). Moreover, some studies demonstrated that PD patients with stiffness had a higher incidence of dyskinesia than tremor (8).

Dyskinesia is unfavorable for quality of life, sometimes being more disabling than PD itself (9, 10). Lower doses and more frequent administration of levodopa may reduce dyskinesia in some patients. However, parkinsonian symptoms and motor fluctuations became worse with the reduction of L-dopa in many cases (11). So patients were encountered with the difficult choice between accepting more serious dyskinesia with better control of PD and less dyskinesia but accompanied by a worsening of PD symptoms. Consequently, it is critical to focus on more effective strategies in order to reduce dyskinesia in the on-state.

Deep brain stimulation (DBS), officially approved by the FDA in 2002, has been proven to improve motor symptoms and dyskinesia. STN and GPi are the two most commonly selected targets. Increasing evidence from randomized clinical trials indicated that the STN DBS and GPi DBS are equally effective in improving motor symptoms and suggests the same in improving dyskinesia (12–15). However, there has been discrepancy as to dyskinesia reduction between two targets. Several randomized controlled trials (RCTs) demonstrated that dyskinesia reduction from GPi DBS was superior to STN DBS (16), whereas other studies indicated there was no significant difference between two targets (17, 18). Up to now, it still remains inconclusive about which target stimulation is more effective in reducing dyskinesia. In the present study, we performed this meta-analysis to evaluate the efficacy of STN and GPi-DBS in the dyskinesia.

## Methods

### Search Strategy and Selection Criteria

A systematic search for articles written in English was performed in PubMed, Cochrane library, and Embase databases according to PRISMA guidelines (19). Databases were searched from inception to January 2018. Medical Subject Headings (MeSH) terms and corresponding keywords were exploded in the electronic search process. The search terms were (MeSH exp Parkinson Disease, and keywords Idiopathic Parkinson's disease, Primary Parkinsonism), (MeSH exp Deep Brain Stimulation and keywords Electrical Stimulation of the Brain and Deep Brain Stimulations), and (MeSH exp Dyskinesias and keywords Dyskinesia). We also examined reference lists of all eligible studies and reviews in the field for further possible titles. The process was repeated until no new titles were found.

**Figure 1:**
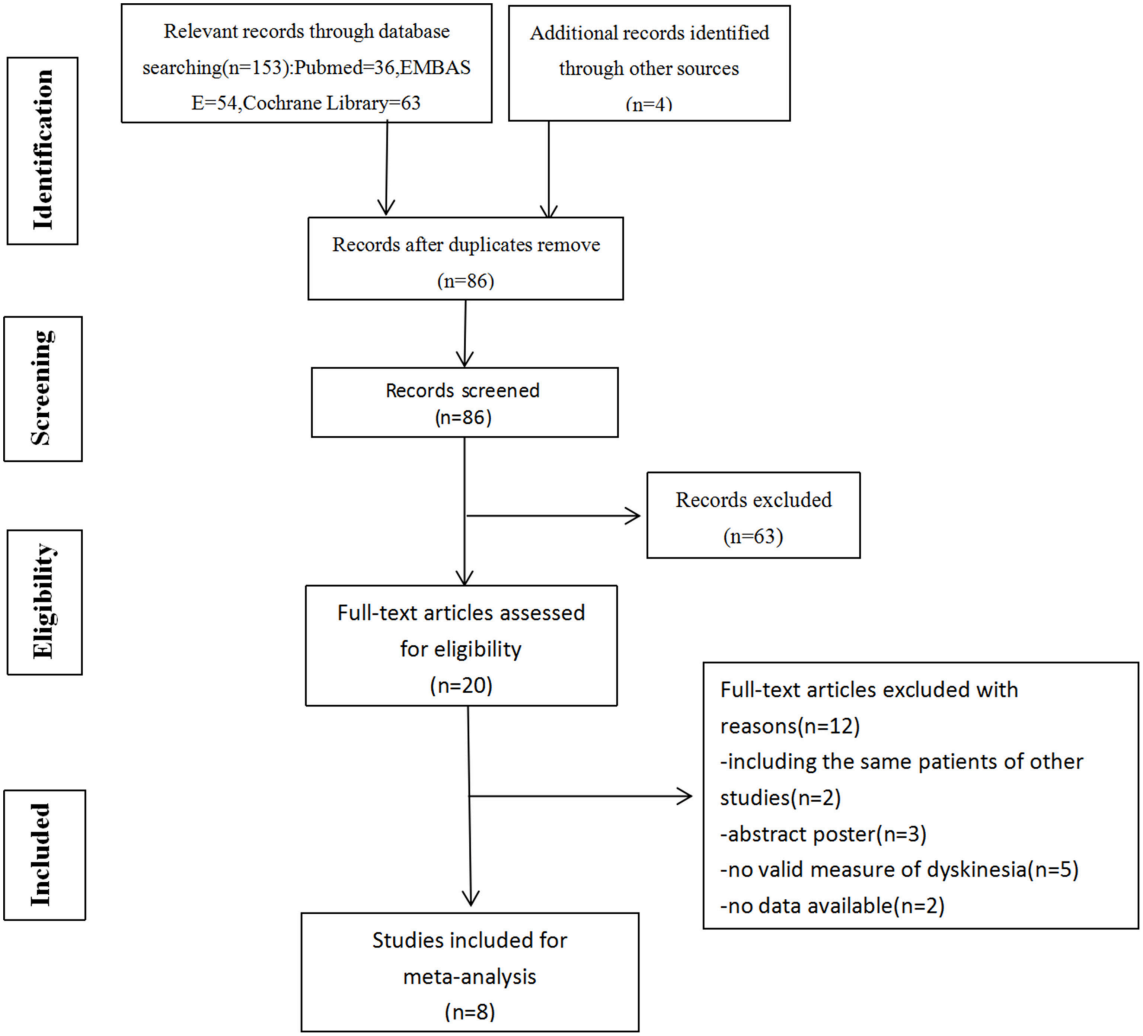
Flow diagram of selection of studies. Among 157 articles screened, 5 randomized controlled studies and 3 non-randomized controlled trials were included in our meta-analysis.

The initial search was conducted by two reviewers independently (YL and FL). Retrieved literatures were imported into endnote, with duplication discarded. Unrelated literatures were excluded after scanning of titles and abstracts carefully. Full-text articles of the remaining literatures were acquired to identify eligibility. Any discrepancy was resolved by discussion or decided by a third reviewer (HL). The PRISMA statement flow diagram displayed the process of literature search and selection, as shown in Figure 1. Published studies were included by meeting the following criteria: (1) population: patients with PD were responsive to levodopa; (2) intervention: GPi DBS or STN DBS (either bilateral or unilateral); (3) comparison: STN DBS or GPi DBS (either bilateral or unilateral); (4) reporting clinical data of dyskinesia before and after surgery. Literatures were excluded for the following reasons: (1) maximum follow-up time < 6 months; (2) data from conference abstracts or literatures that could not be extracted.

### Data Extraction

Key characteristics of studies were extracted independently by two authors (QH and LC), ready for comparative analysis. All data were tabulated onto a predefined spreadsheet. For each included study, the following were extracted: authors, title, journal, year of publication, participant characteristics, dyskinesia scores, LED scores, and assessment time points in relation to DBS.

### Data Analysis

Data analysis was performed by the RevMan 5.3 (The Cochrane Collaboration, London, UK). All the outcomes were displayed in consistent data. Standardized mean differences (SMD) with 95% confidence intervals (CI) were calculated for dyskinesia, since the analyzed domains involved multiple testing instruments. Mean differences (MD) with 95% CI were reported for the LED. The heterogeneity across studies was calculated using I-square and chi-square. Once the heterogeneity was small (*I*^2^ < 50%), the fixed-effects model was used; otherwise, the random effects model was used. *P* < 0.05 was considered statistically significant.

### Quality Assessment

The methodological quality of the selected studies was assessed using Cochrane collaboration's tool. The risk of bias tool included six domains: selection, performance, detection, attrition, reporting and other bias (20). Methodological Index for Non-randomized Studies (MINORS) was used for assessing the quality of non-randomized controlled studies. MINORS involved 12 items for comparative studies, subsequently each item was scored from 0 to 2; 0 indicating that it was not reported in the article, 1 indicating that it was reported but inadequately, and 2 indicating that it was reported adequately (21).

## Results

### Study Characteristics

Initially, we identified 157 articles, 86 of which remained after removal of duplicates. A total of 20 full-text articles were assessed for eligibility, 5 randomized controlled studies and 3 non-randomized controlled trials were included in our meta-analysis at the end. Totally, 822 patients were included, among which 453 had been implanted with STN DBS, 369 with GPi DBS. The characteristics of the studies were presented in Table 1.

**Table 1:** Characteristics of included controlled trials.

**Study**	**Targets**	**Surgical modus**	**Subject size, *n***	**Age (years)**	**Disease duration (years)**	**Outcome measure**	**Assessment time points**
Anderson et al. (22)	STN	Bilateral	12	61.0 ± 9.0	15.6 ± 5.0	Dyskinesia severity rating	Baseline, 12 months
	GPi		11	54.0 ± 12.0	10.3 ± 2.0		
Burchiel et al. (18)	STN	Bilateral	6	62.8 ± 12.0	13.6 ± 5.0	Dyskinesia severity rating	Baseline, 12 months
	GPi		4	46.5 ± 11.0	10.6 ± 2.0		
Rodriguez-Oroz et al. (23)	STN	Bilateral	49	59.8 ± 9.8	14.1 ± 5.9	A dyskinesia scale	Baseline, 12 months 3-4y
	GPi		20	55.8 ± 9.4	14.4 ± 5.7	LED	
Odekerken et al. (16)	STN	Bilateral	63	60.9 ± 7.6	12.0 ± 5.3	CDRS	Baseline, 12 months
	GPi		65	59.1 ± 7.8	10.8 ± 4.2	LED	
Nutt et al. (24)	STN	Bilateral	6	56.5 ± 15.1	9.5 ± 2.2	Dyskinesia severity rating	Baseline, 12 months
	GPi		6	56.8 ± 11.5	19.5 ± 3.9		
Follett et al. (17)	STN	Bilateral	147	61.9 ± 8.7	11.1 ± 5.0	Motor function with Dyskinesia LED	Baseline, 24months
	GPi		152	61.8 ± 8.7	11.5 ± 5.4		
Weaver et al. (25)	STN	Bilateral	70	60.7 ± 8.9	11.3 ± 4.7	Motor function with Dyskinesia LED	Baseline, 6 months 24 month, 36 months
	GPi		89	60.4 ± 8.3	11.4 ± 4.9		
Obeso et al. (26)	STN	Bilateral	96	59.0 ± 9.6	44.6 ± 8.9	Motor function with Dyskinesia LED	Baseline, 6 months
	GPi		38	55.7 ± 9.8	41.2 ± 9.5		

*CDRS, clinical dyskinesia rating scale; DBS, deep brain stimulation; GPi, globus pallidus interna; LED, levodopa equivalent doses; STN, subthalamic nucleus; UPDRS IV, unified Parkinson's disease rating scale IV*.

### Study Quality

**Figure 2:**
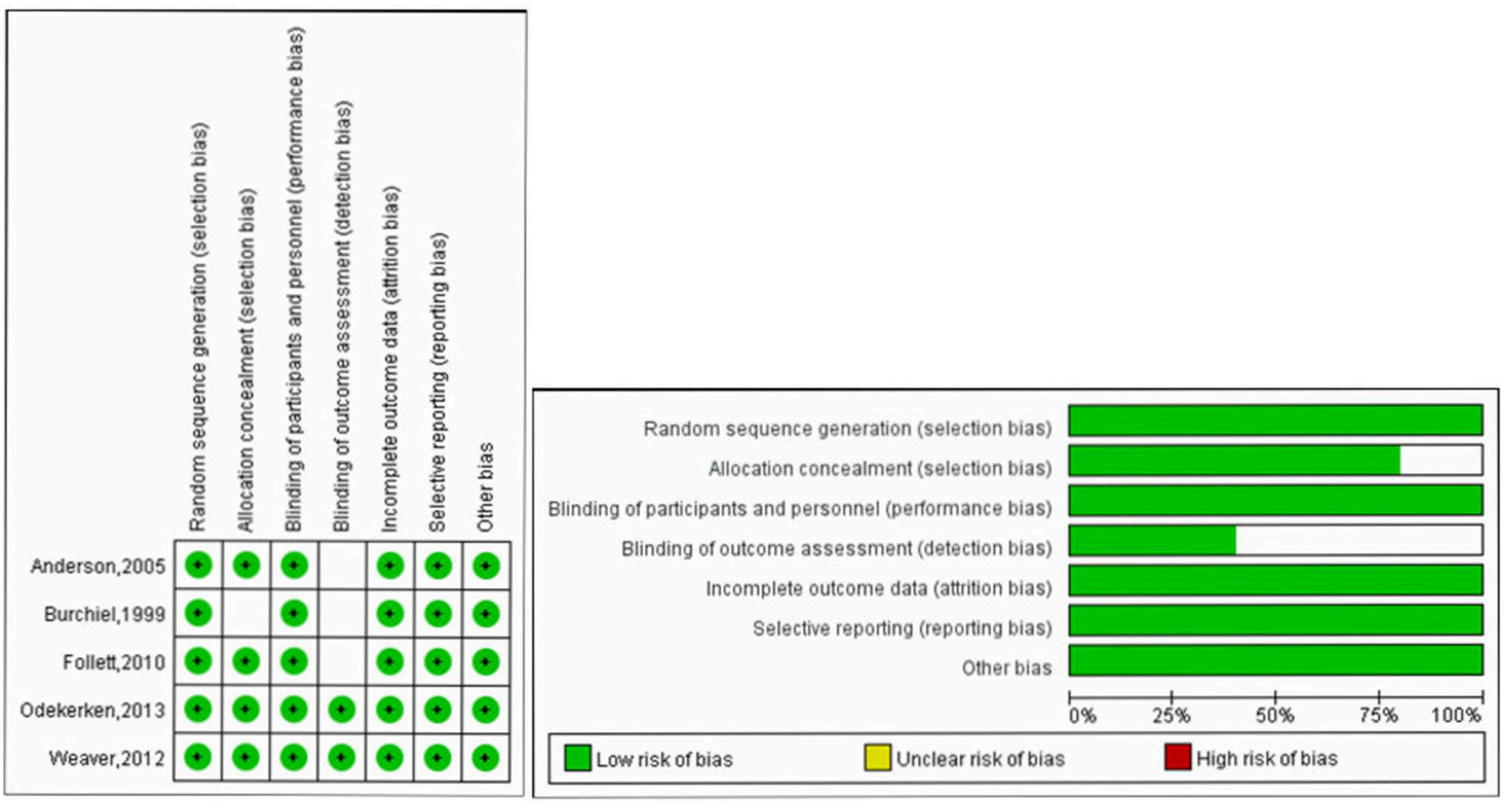
Quality assessment of RCTs using Cochrane collaboration's tool for assessing risk of bias.

Study quality of RCTs was evaluated by Cochrane collaboration's tool, Two RCTs were classified as high quality (16, 25), and the other three RCTs were classified as moderate quality (17, 18, 22). Quality assessment results were presented in Figure 2. For the other three cohort studies evaluated by Methodological Index for Non-randomized Studies (MINORS), two studies (24, 26) scored 16 points and one study (23) scored 18 points, which could be regarded as at moderate-quality (Table 2). Thus, all included studies were deemed to be of the moderate or high quality. Most RCTs lost points because of the lack of blinding and allocation concealment. While most cohort studies lost points because of a statement of the outcome of interest at the beginning and non-blind outcome assessment.

**Table 2:** Risk of bias results assessed with methodological index for non-randomized studies (MINORS).

**Study**	**A**	**B**	**C**	**D**	**E**	**F**	**G**	**H**	**I**	**J**	**K**	**L**	**Total score**
Rodriguez-Oroz et al. (23)	2	2	2	2	0	2	2	0	2	2	0	2	18
Nutt et al. (24)	2	0	2	2	0	2	2	0	2	2	0	2	16
Obeso et al. (26)	2	0	2	2	0	2	2	0	2	2	0	2	16

*A, a stated aim of the study; B, inclusion of consecutive patients; C, prospective collection of data; D, endpoint appropriate to the study aim; E, unbiased evaluation of endpoints; F, follow-up period appropriate to the major endpoint; G, loss to follow-up not exceeding 5%; H, prospective calculation of the study size; I, an adequate control group; J, contemporary groups; K, baseline equivalence of groups; L, adequate statistical analyses*.

### Profile Comparison

Meta-analysis results of pretreatment profiles were shown in Table 3. Significant heterogeneity was detected in duration of disease (*I*^2^ = 87%) and dyskinesia (*I*^2^ = 46%). The heterogeneity was greatly reduced (*I*^2^ = 0%) when two studies (24, 26) were excluded. A significant difference in pretreatment age was observed in STN DBS group compared with GPI DBS group, with an overall pooled MD of 1.34 (95% CI = [0.12, 2.56]), indicating that the patients with STN DBS were generally older than the patients with GPi DBS. There were no significant differences and heterogeneity in the other comparisons of pretreatment profiles. Forest plots of each comparison were presented in Supplementary Data (S1–S6).

**Table 3:** Meta-analysis results of Profile comparison for STN DBS vs. GPi DBS.

**Item**	***I*^**2**^statistic**	**Mean and 95%CI (fixed-effect model)**	**Mean and 95% CI (randomized-effect model)**
Age	31%	1.34 [0.12, 2.56]	1.78 [0.09, 3.47]
Duration of disease (month)	87%	−0.16 [−0.57, 0.89]	−0.06 [−2.30, 2.17]
LED (mg/day)	39%	−17.34 [−97.39, 62.71]	−2.56 [−109.97, 104.84]
UPDRS off-med	0%	1.58 [−0.34, 3.49]	1.58 [−0.34, 3.49]
UPDRS on-med	0%	0.40 [−1.11, 1.90]	0.40 [−1.11, 1.90]
dyskinesia	46%	−0.10 [−0.24, 0.04]	−0.11 [−0.33, 0.11]

*CI, confidence interval; GPi, globus pallidus interna; LED, Levodopa equivalent doses; STN, subthalamic; UPDRS, unified Parkinson's disease rating score*.

### Changes in Dyskinesia Scores

**Figure 3:**
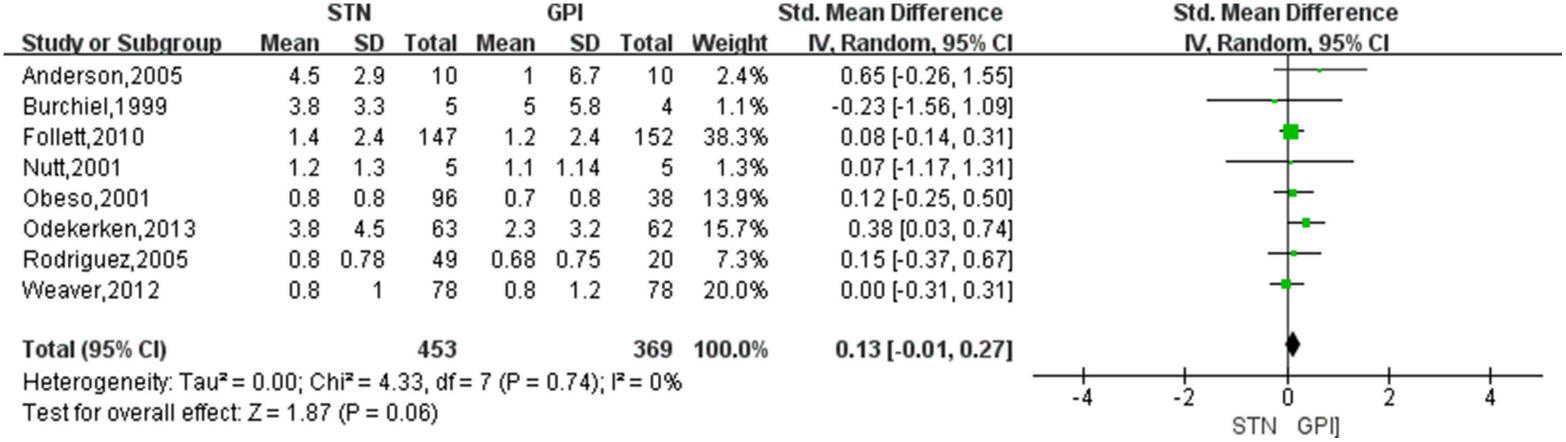
Forest plot of mean difference of dyskinesia score in the on-medication/on-stimulation state between STN DBS and GPi DBS. GPi, globus pallidus interna; STN, subthalamic nucleus; IV, inverse variance; CI, confidence interval; Std, standardized.

**Figure 4:**
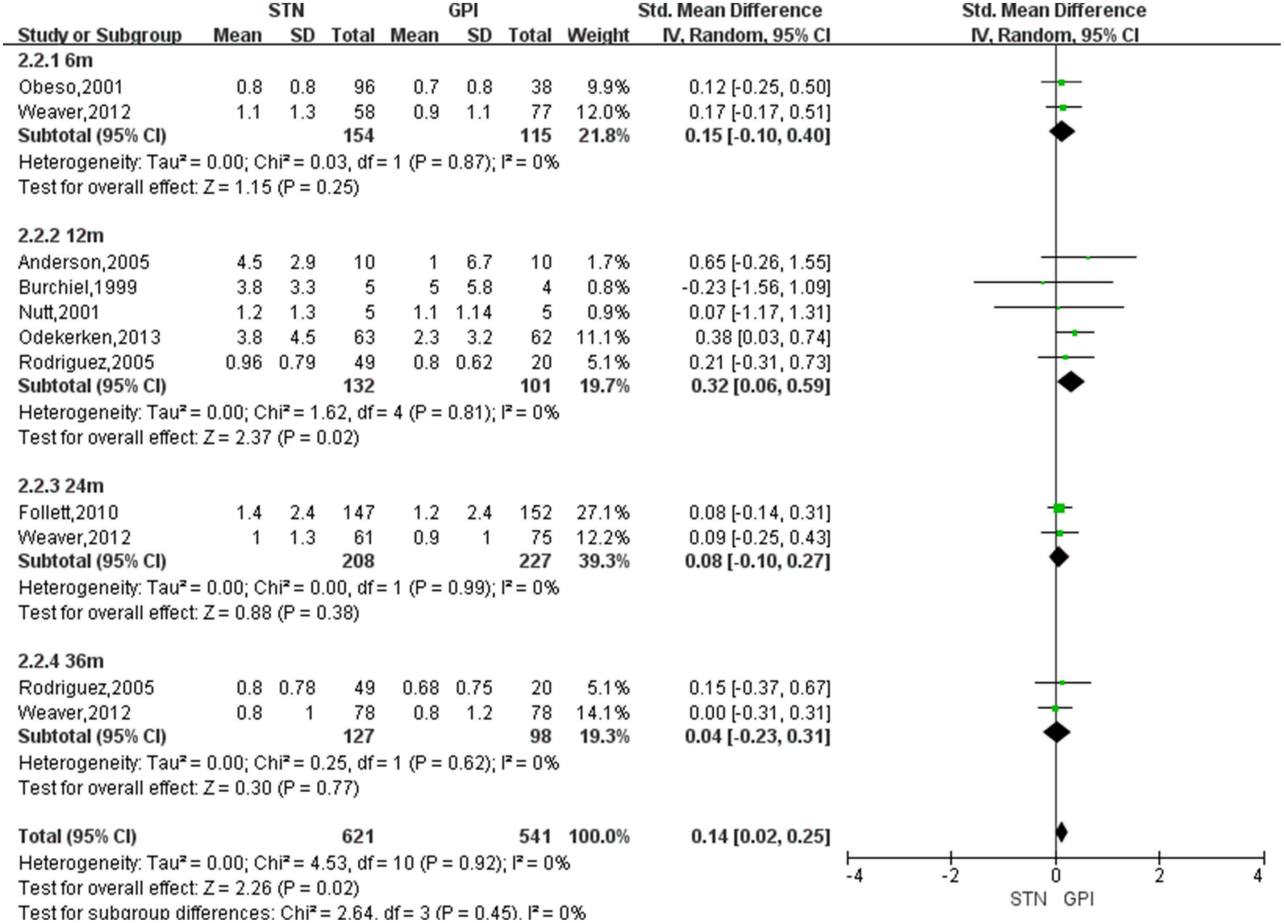
Forest plot: subgroup analyses were conducted according to follow-up periods in dyskinesia score between STN DBS and GPi DBS. GPi, globus pallidus interna; STN, subthalamic nucleus; IV, inverse variance; CI, confidence interval; Std, standardized.

Based on the results of meta-analysis, GPi DBS did not yield any significant improvement in the dyskinesia score over STN DBS, with a change score of 0.13 (95% CI = −0.01 to 0.27, *P* = 0.006; Figure 3). No significant differences in heterogeneity was observed between treatment groups (*X*^2^ = 4.33, df = 7, *p* = 0.74, *I*^2^ = 0%). We conducted subgroup analyses according to follow-up periods. A greater reduction of dyskinesia was observed in GPi DBS group compared with STN DBS group at 12 months after surgery, with an overall pooled SMD of 0.32 (95% CI = 0.06 to 0.59, *P* = 0.02; Figure 4), with evidence of no heterogeneity (*X*^2^ = 1.62, df = 4, *p* = 0.81, *I*^2^ = 0%). However, no significant differences and heterogeneity were observed in the other follow-up periods.

### Changes in LED Scores

**Figure 5:**
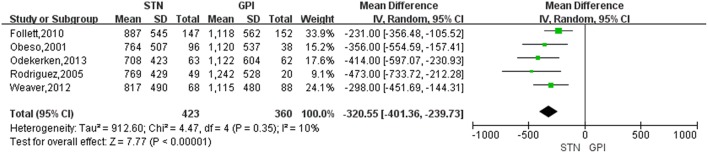
Forest plot of standardized mean difference of levodopa equivalent doses between STN DBS and GPi DBS. GPi, globus pallidus interna; STN, subthalamic nucleus; IV, inverse variance; CI, confidence interval.

**Figure 6:**
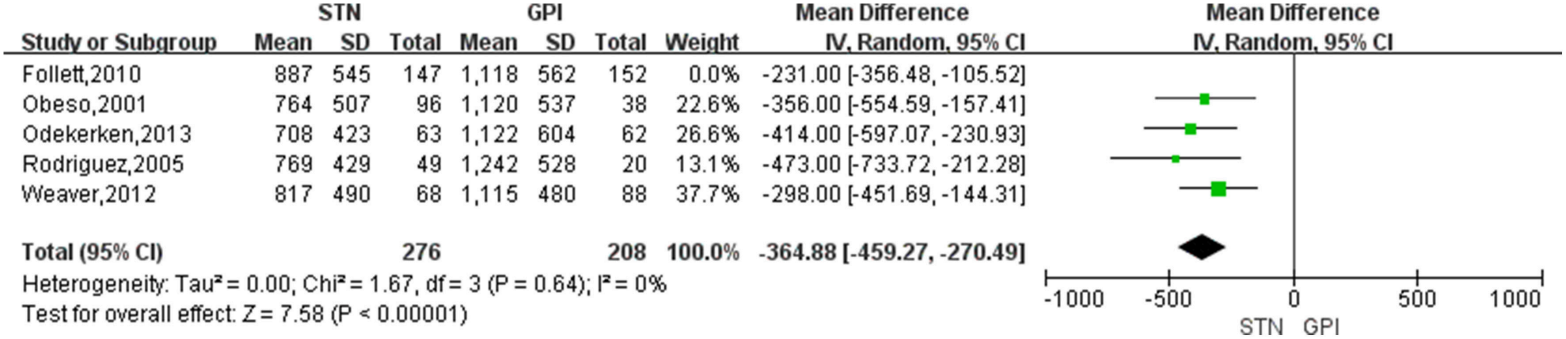
Forest plot: sensitivity analysis. GPi, globus pallidus interna; STN, subthalamic nucleus; IV, inverse variance; CI, confidence interval.

Treatment of STN DBS was associated with a greater reduction of LED compared with GPi DBS, with a change score of −320.55 (95% CI = −401.36 to −239.73, *P* < 0.00001; Figure 5). Based on the Chi-square and I-square analyses, there was small difference in heterogeneity between treatment groups (*X*^2^ = 4.47, df = 4, *p* = 0.35, *I*^2^ = 10%). The heterogeneity was greatly reduced (*I*^2^ = 0%) when two studies (17, 24) were exclude [Figure 6, for example, the study by Follett et al. (17) was excluded]. However, even after excluding one or the other of those studies, LED were still reduced to a greater extent after STN DBS than GPi DBS.

### Publication Bias

Publication bias was estimated by funnel plots. No obvious asymmetry was identified in funnel plots, indicating that there was no publication bias (Supplementary Data S7).

## Discussion

The current meta-analysis provides a review of the efficacy of STN and GPi DBS in the dyskinesia in the treatment of advanced PD. Eight controlled clinical trials were included in this meta-analysis. Changes in dyskinesia scores and LED scores from baseline values after DBS were used to assess improvements in dyskinesia and medication use in patients with PD. Our findings revealed that there was a greater reduction of dyskinesia scores from GPi DBS compared with STN DBS at 12 months follow-up. There were two randomized clinical trials that revealed that GPi stimulation was superior in dyskinesia reduction to STN stimulation at 12 months after surgery (16, 22). However, there was no statistically significant difference between GPi DBS and STN DBS in the other follow-up periods, which was consistent with the VA Cooperative Study (25). Furthermore, STN DBS allowed for medication dosages to be reduced to lower levels than GPi DBS. Therefore, our results indicated that GPi DBS offered a greater reduction of dyskinesia than STN DBS at 12 months after surgery.

DBS has been established as an important therapeutic strategy to relieve motor symptoms in advanced PD patients when motor symptoms are no longer managed adequately with levodopa treatment (27). STN and GPi are the two most commonly selected targets. Moreover, mounting evidence has confirmed similar effect of the two targets stimulation on improvement of motor function and dyskinesia observed in several meta-analyses of RCTs involved in DBS therapy (28–30). Nevertheless, the mechanisms of dyskinesia reduction in STN and GPi DBS are fundamentally different. GPi stimulation improved dyskinesia through direct stimulation effects on dopaminergic pathways to inhibit abnormal electrical activity of GPi (22, 31, 32), while STN stimulation reduced dyskinesia by lowering greater dopaminergic medication to minimize dyskinesia (16, 33). Further investigations are needed to focus on the exact mechanisms of dyskinesia changes after stimulation of the two targets.

Targets election for DBS should be assessed based on the patient's specific characteristics and goals. Deep brain stimulation of the STN is advantageous if the main goal is dopaminergic medication reduction. However, medication reduction may aggravate depression and apathy, even increase suicidal ideation (34). GPi stimulation rather than STN stimulation can be considered in patients with cognitive decline or mood changes (25). In patients with prominent gait disorder, axial symptoms, or falls, GPi DBS may be preferable (35). Successful GPi DBS was also applied in cases of persistent or severe dyskinesia, especially if they were unable to sufficiently reduce dopaminergic treatment (36).

The changes in LED observed in our analysis were consistent with the results of the meta-analysis (29) and the outcome of other recent studies, which indicated that medication was markedly reduced after STN DBS compared with GPi DBS. Although medication reduction is not the primary goal of surgery, dopaminergic requirements are reduced, with the additional advantageous of decreased fluctuations in “on” and “off” state, drug-induced dyskinesia, and other complications of medications (28–30). However, the reduction in medication should be managed cautiously, neurosurgeons have to avoid aggressive medication reduction after STN DBS, since apathy, depressive symptoms, and increased suicidal ideation may occur once levodopa was rapidly withdrawn (34). Previous study demonstrated that the loss of prior positive effects of STN stimulation in the medication “on” phase especially for gait and balance was related to a reduction in dopaminergic medication, not observed in GPi-DBS patients which retained stable scores (37). This contributed to various thoughts such as the desirability of medication reduction in the absence of side effects, the relationship between medications and stimulation.

Some limitations should be considered in our study. First, three studies lacked LED data (18, 22, 24). The involved studies were conducted with various implantation techniques, stimulators, stimulation parameters, and postoperative management. Therefore, potential risks of significant heterogeneity were undefined. Both randomized and non-randomized studies were included in the same analysis, which might result in potential bias. However, even after excluding the non-randomized studies, the outcomes were still stable (Supplementary Data S8). Second, the analyzed domains about dyskinesia involved multiple testing instruments, and the measurements in those studies of our meta-analysis were performed in different times after surgery, which might cause bias. Finally, we only included studies published in English, which might result in potential bias.

## Conclusion

GPi DBS is superior to reduce dyskinesia than STN DBS at 12 months after surgery for advanced PD patients, and the mechanisms of dyskinesia reduction in STN and GPi DBS are fundamentally different. STN DBS allowed for significant dopaminergic medication reduction. Further studies should focus on the different mechanism for dyskinesia reduction by GPi or STN DBS.

## Author Contributions

YL and ZX: conception and design, drafting the article. QH and LC: acquisition of data. YL, FL, and HL: analysis and interpretation of data. ZX: approved the final version of the manuscript on behalf of all authors. ZX, WZ, YC, LC, and YL: study supervision. All authors critically revising the article, reviewed submitted version of manuscript.

### Conflict of Interest Statement

The authors declare that the research was conducted in the absence of any commercial or financial relationships that could be construed as a potential conflict of interest.
