# Supplementary material for: Improvement of Deep Brain Stimulation in Dyskinesia in Parkinson's Disease: A Meta-Analysis
Source: Front Neurol. 2019 Feb 25;10:151. doi: 10.3389/fneur.2019.00151 (PMC6397831; doi:10.3389/fneur.2019.00151)
Supplement: Supplementary file 1 [file Data_Sheet_1.docx]

S1 Forest plot of mean difference of Age of pretreatment profiles between STN DBS and GPi DBS


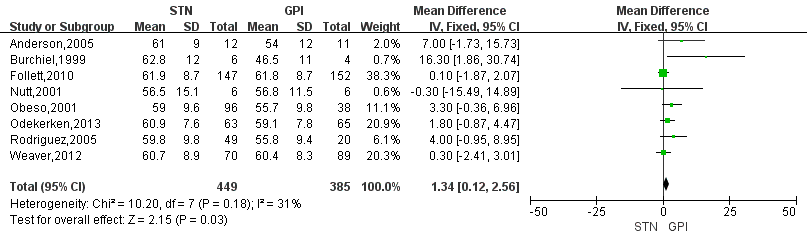


Individual MD and their corresponding 95% confidence intervals were indicated by filled squares. The size of the square indicated the weight of the study in the fixed effect meta-analysis. The summary estimate of MD and its 95% confidence interval were indicated by a diamond. GPi, globus pallidus interna; STN, subthalamic nucleus; IV, inverse variance; CI, confidence interval.

S2 Forest plot of mean difference of Duration of disease (month) of pretreatment profiles between STN DBS and GPi DBS


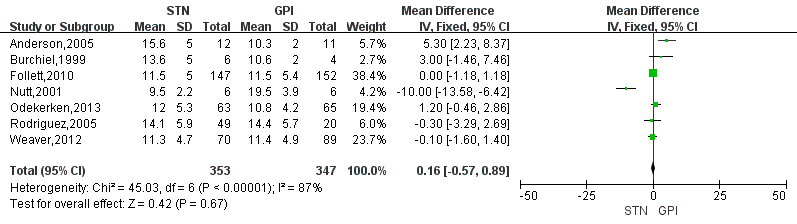
Individual MD and their corresponding 95% confidence intervals were indicated by filled squares. The size of the square indicated the weight of the study in the fixed effect meta-analysis. The summary estimate of MD and its 95% confidence interval were indicated by a diamond. GPi, globus pallidus interna; STN, subthalamic nucleus; IV, inverse variance; CI, confidence interval.

S3 Forest plot of mean difference of LED (mg/day) of pretreatment profiles between STN DBS and GPi DBS


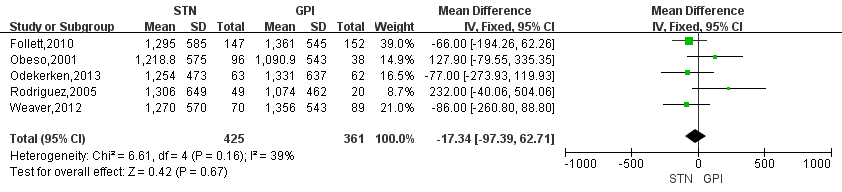
Individual MD and their corresponding 95% confidence intervals were indicated by filled squares. The size of the square indicated the weight of the study in the fixed effect meta-analysis. The summary estimate of MD and its 95% confidence interval were indicated by a diamond. GPi, globus pallidus interna; STN, subthalamic nucleus; IV, inverse variance; CI, confidence interval.

S4 Forest plot of mean difference of UPDRSⅢ off-med of pretreatment profiles between STN DBS and GPi DBS


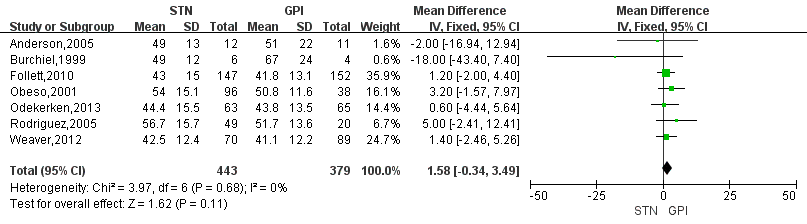
Individual MD and their corresponding 95% confidence intervals were indicated by filled squares. The size of the square indicated the weight of the study in the fixed effect meta-analysis. The summary estimate of MD and its 95% confidence interval were indicated by a diamond. GPi, globus pallidus interna; STN, subthalamic nucleus; IV, inverse variance; CI, confidence interval.

S5 Forest plot of mean difference of UPDRSⅢ on-med of pretreatment profiles between STN DBS and GPi DBS


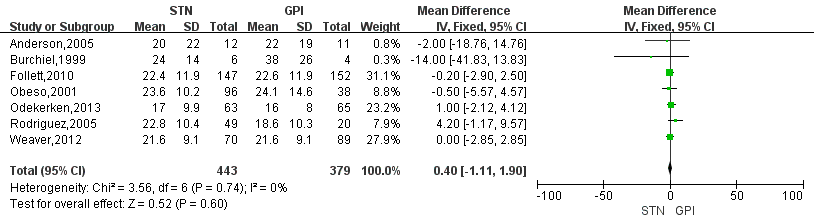
 Individual MD and their corresponding 95% confidence intervals were indicated by filled squares. The size of the square indicated the weight of the study in the fixed effect meta-analysis. The summary estimate of MD and its 95% confidence interval were indicated by a diamond. GPi, globus pallidus interna; STN, subthalamic nucleus; IV, inverse variance; CI, confidence interval.

S6 Forest plot of mean difference of dyskinesias of pretreatment profiles between STN DBS and GPi DBS


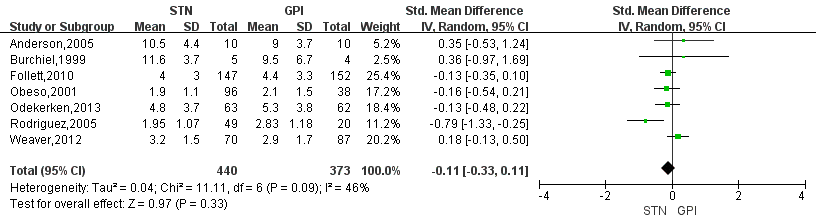
 Individual SMD and their corresponding 95% confidence intervals were indicated by filled squares. The size of the square indicated the weight of the study in the random effect meta-analysis. The summary estimate of SMD and its 95% confidence interval were indicated by a diamond. GPi, globus pallidus interna; STN, subthalamic nucleus; IV, inverse variance; CI, confidence interval.

S7 funnel plots of comparison for dyskinesias


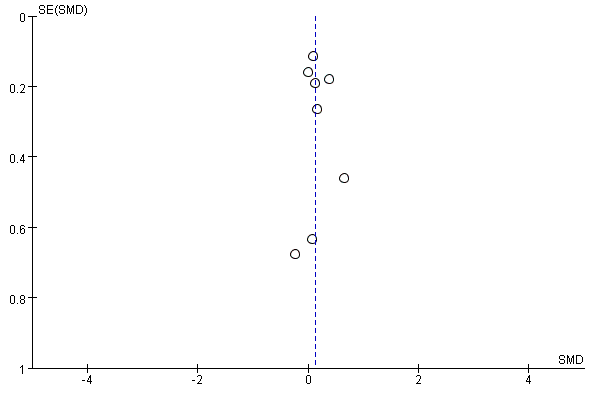


The funnel plot appeared symmetric. Each small circle represents an independent study for the indicated association.

S8 funnel plots of excluding the non-randomized studies


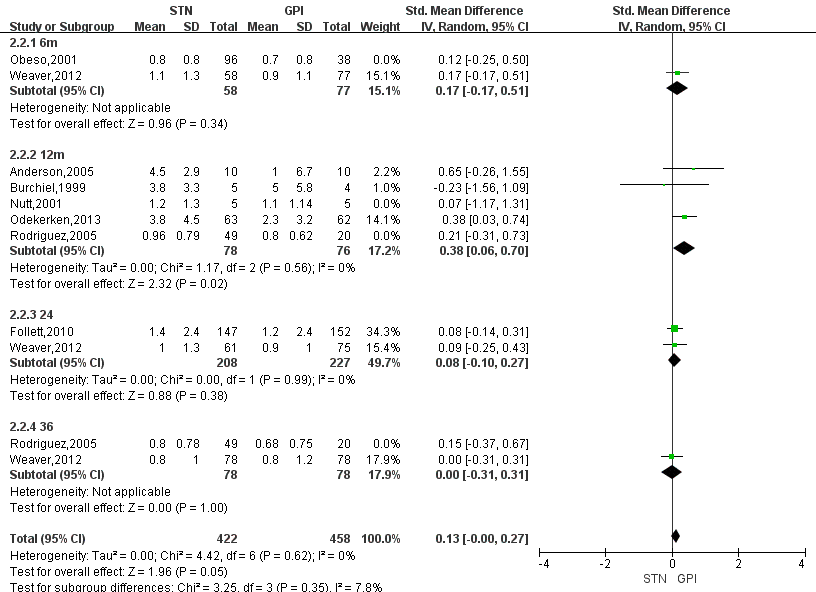


Abbreviations: GPi, globus pallidus interna; STN, subthalamic nucleus；IV, inverse variance; CI, confidence interval；Std, standardized.
